# Anti-Periodontopathogenic Ability of Mangrove Leaves ( *Aegiceras corniculatum* ) Ethanol Extract: *In silico* and *in vitro* study

**DOI:** 10.1055/s-0041-1741374

**Published:** 2022-04-22

**Authors:** Alexander Patera Nugraha, Mada Triandala Sibero, Albertus Putera Nugraha, Martining Shoffa Puspitaningrum, Yuniar Rizqianti, Desintya Rahmadhani, Viol Dhea Kharisma, Nastiti Faradilla Ramadhani, Rini Devijanti Ridwan, Tengku Natasha Eleena binti Tengku Ahmad Noor, Diah Savitri Ernawati

**Affiliations:** 1 Department Orthodontics, Faculty of Dental Medicine, Universitas Airlangga, Surabaya, Indonesia; 2 Division of Dental Health Science, Faculty of Dental Medicine, Universitas Airlangga, Surabaya, Indonesia; 3 Marine Science Department, Faculty of Fisheries and Marine Science, Diponegoro University, Semarang, Indonesia; 4 Faculty of Medicine, Universitas Airlangga, Universitas Airlangga, Surabaya, Indonesia; 5 Faculty of Dental Medicine, Universitas Airlangga, Universitas Airlangga, Surabaya, Indonesia; 6 Department of Biology, Faculty of Mathematic and Natural Science, Universitas Brawijaya, Surabaya, Indonesia; 7 Dentomaxillofacial Department, Faculty of Dental Medicine, Universitas Airlangga, Surabaya, Indonesia; 8 Department of Oral Biology, Faculty of Dental Medicine, Universitas Airlangga, Surabaya, Indonesia; 9 609 Armed Forces Dental Clinic, Kem Semenggo, Kuching, Sarawak, Malaysia; 10 Department of Oral Medicine, Faculty of Dental Medicine, Universitas Airlangga, Surabaya, Indonesia

**Keywords:** mangrove leaf extract, human health, good health and well-being, medicine, dentistry

## Abstract

**Objective** Mangrove ( *Aegiceras corniculatum* ) is an abundant natural marine resource of Indonesia, which can be explored for treating periodontal disease due to its potential as immunoregulatory, antibacterial, and antioxidant properties. The objective of this study was to investigate the active compound from Indonesian mangrove leaf extract ( *A. corniculatum* ) (MLE) for developing a herbal-based mouthwash through *in silico* and *in vitro* studies.

**Materials and Methods** Phytochemistry and liquid chromatography-high resolution mass spectrometry (LC-HRMS) were done to explore the active compounds in MLE. Chemistry screening and interaction, absorption, distribution, metabolism, and excretion (ADME), molecular docking simulation, and visualization of MLE active compounds as anti-inflammatory, antioxidant, and antibacterial were investigated *in silico* The inhibition zone of MLE against *Aggregatibacter actinomycetemcomitans* (Aa), *Porphyromonas gingivalis* (Pg), and *Fusobacterium nucleatum* (Fn) as periodontopathogenic bacterias was performed by diffusion method. Doxycycline 100 mg was used as a positive control, as a treatment group, there were five groups, namely 0%, 25%, 50%, 75%, and 100% MLE.

**Results** Alkaloid, saponin, flavonoid, triterpenoid, steroid, tannin, and quinone were detected in MLE. A high concentration of (-)epicatechin and coumaric acid (CA) were found in MLE. MLE in 100% concentration has the most effective ability to inhibit *Fn, Pg, Aa* growth *in vitro* . (-)-Epicatechin has a higher negative binding affinity than CA that can enhance heat shock protein (HSP)-30, HSP-70, HSP-90, interleukin-10, and FOXP3 and also inhibit interleukin-6, peptidoglycan, flagellin, and dectin *in silico* .

**Conclusion** MLE of *A. corniculatum* has antioxidant, anti-inflammatory, and antibacterial activities that can be a potential raw material for developing a herbal-based mouthwash.

## Introduction

Periodontitis is a multifactorial disease that causes inflammation of the periodontal tissues. Chronic periodontitis is induced by microorganisms that stimulate the host immune and inflammatory responses. 1 2 The recent Global Burden of Disease Study indicates that severe periodontitis is the sixth most prevalent disease worldwide, with an overall prevalence of 11.2% and around 743 million people affected, and the global burden of periodontal disease increased by 57.3% from 1990 to 2010. 3 Periodontal disease, especially between its mild and moderate forms, is highly prevalent in adult-aged populations all over the world, with prevalence rates around 50%, while its severe form increases especially between the third and fourth decades of life, with the global prevalence being around 10%. 4 Based on Riset Kesehatan Dasar (RISKESDAS) or Basic Health Research in 2018, the percentage of periodontitis cases in Indonesia is 74.1%. 5

Periodontitis may occur because of the complex interaction between the subgingival biofilm and the host immune response that develops in the gingival and periodontal tissues in response to a bacterial attack. 6 Some pathogenic bacteria species that have been associated with the development of periodontal disease are *Aggregatibacter actinomycetemcomitans (Aa), Porphyromonas gingivalis (Pg), and Fusobacterium nucleatum (Fn)* . The presence of bacteria within gingival tissue following invasion leads to the infiltration of a few inflammatory cells. In addition, dental plaque provides a source of continuously invading bacteria as a reservoir. Such persistent infection leads to chronic inflammation and tissue destruction. 4 7 8

Periodontal disease has a significant impact on oral health-related quality of life (OH-QoL), especially with the worsening and extension of the disease in which it presents higher destructive consequences. 9 As periodontitis is the major cause of tooth loss in the adult population worldwide, these individuals are at risk of multiple tooth loss, edentulism, and masticatory dysfunction, thereby affecting their nutrition, quality of life, and self-esteem as well as imposing huge socio-economic impacts and healthcare costs. 3 Treatment of periodontitis is important to eliminate infections around the dental tissue and also to improve systemic health in general. 2

Periodontal treatment is performed using nonsurgical and surgical periodontal therapies. 10 The nonsurgical therapy of periodontitis is performed by scaling root planing (SRP) and debridement to remove biofilm and calculus from the surface, but there are limitations in this treatment, there is the inability to access the pocket area and root furcation. 11 The use of antibiotics (local or systemic antimicrobial) has been proposed to reduce the number of microorganisms and consequently minimize the host response related to tissue destruction. 10 The majority of the population may not perform mechanical plaque removal sufficiently. Thus, antimicrobial mouth rinses may be advantageous for the prevention and treatment of periodontitis. 12 Mouthwash is an option to maintain plaque control and treatment after periodontal therapy. 13 Chlorhexidine (CHX) digluconate is considered to be one of the most frequently used compounds for mouthwash. In contrast, the side effects of CHX include taste alteration, brown discoloration of teeth, restorative materials, and dorsum of the tongue. 14

Natural materials are currently being developed in treating periodontitis. As a maritime country, Indonesia has coastal potential herbal resources that can be used as a source of active ingredients in a mouthwash. One of the coastal plants that has not been studied much is mangroves. However, various mangrove species from the general Avicennia, Bruguiera, Rhizophora, and Xylocarpus can be found in Asia. 15 16 17 One of the mangrove species that is rarely studied in Indonesia is *Aegiceras corniculatum* . Studies that have been conducted using this mangrove extract are as blood anticoagulant, antiplasmodial, and anti-inflammatory agents. 18 19 20 Moreover, various reports have stated that *A. corniculatum* has antibacterial activity against human pathogens such as *Escherichia coli, Klebsiella pneumoniae, Mycobacteria tuberculosis, Staphylococcus aureus, Vibrio harveyi, and V. parahaemolyticus* . 21 22 23 This shows that this plant has various health potentials, especially as a producer of antimicrobial compounds. Based on these results, it is suspected that *A. corniculatum* is also capable of producing antimicrobial compounds that can be used as active ingredients in mouthwash formulations to prevent periodontal disease.

Molecular docking is a computational method used to predict the interaction of two molecules generating a binding model. In many drug discovery applications, docking is done between a small molecule and a macromolecule, for example, protein-ligand docking. More recently, docking is also applied to predict the binding mode between two macromolecules, for instance, protein–protein docking. 24 Several compounds in *A. corniculatum* have been found to have potential as an anti-inflammatory and anti-microbial effect. The hypothesis of this study is *A. corniculatum* from Indonesia may possess anti-periodontopathogenic ability *in silico and in vitro.* Furthermore, in this study, the active compound from Indonesian mangrove leaf extract ( *A. corniculatum* ) (MLE) was investigated for developing a herbal-based mouthwash through *in silico* and in vitro studies.

## Materials and Methods

The ethical clearance for this study was obtained from Universitas Airlangga Surabaya, Faculty of Dental Medicine Health Research Ethical Clearance Commission with number: 494/HRECC/FODM/VIII/2021.

### *In silico* Study

#### Sample Retrieval and Absorption, Distribution, Metabolism, Excretion-Toxicology analysis

Chemical compounds containing mangrove extract used in this study consisted of (-)-epicatechin, coumaric acid, and ascorbic acid obtained from PubChem ( https://pubchem.ncbi.nlm.nih.gov/ ). The samples were identified by ID, formula, weight, and canonical smile, then the ligand structure was minimized in the PyRx software to increase the flexibility. 25 Preparations were performed on The Research Collaboratory for Structural Bioinformatics Protein Data Bank (RCSB PDB) ( https://www.rcsb.org/ ) proteins consisting of HSP70, HSP30, HSP90, IL10, Foxp3, IL-6, peptidoglycan, flagellin, and dectin, information on target proteins was obtained from the database and consisted of ID, visualization method, resolution, atomic count, weight, chain, and sequence length. Next, sterilization of protein samples was performed using the PyMol software for molecular docking optimization. 26 Druglikeness analysis was performed on the three chemical compounds containing mangrove with reference to Lipinski rules ( http://www.scfbio-iitd.res.in/software/drugdesign/lipinski.jsp ) and Absorption, Distribution, Metabolism, Excretion-Toxicology (ADME-Tox) analysis ( http://www.swissadme.ch/ ).

#### Molecular Docking Simulation

The activity of ligand binding to the target protein domain was identified by molecular docking simulation. The aim was to determine the type of activity—inhibitory or enhancing the activity of the target. The binding energy produced by the ligand when it binds to the target protein site can trigger a specific biological response, the more negative the binding score, the higher the effect on target protein activity. 27 The ligands in this study were three compounds containing mangroves and target proteins consisting of HSP70, HSP30, HSP90, IL10, Foxp3, IL-6, peptidoglycan, flagellin, and dectin.

#### Ligand–Protein Interaction

Identification of the type of activity of the target protein triggered by the two ligands is known through the analysis of the position and chemical bonds in the molecular complex resulting from molecular docking in the Discovery Studio software. Hydrophobic, hydrogen, pi, and Van der Waals bonds formed in molecular complexes can be identified through the server, the type of weak bond interactions can play a role in the context of the biological activity of a protein. 28 29

#### Molecular Visualization

This research used the PyMol software for three-dimensional (3D) visualization of molecular docking results. The 3D structure of the ligand–protein molecular complex is shown in the form of cartoons, surfaces, sticks, and spheres. 30

#### Sample Collection, Identification, and Preparation

Mangrove samples were taken from MECoK Ecopark, Diponegoro University Campus in Jepara. Fresh mangrove leaves without any indication of damage (physical or disease) were collected by picking and then stored in dark plastic samples and stored in a cool box to prevent damage to metabolites due to light and temperature. The leaves, stems, roots, and flowers were collected to serve as the basis for identification keys. The samples obtained were then prepared at the Natural Product Laboratory, Diponegoro University. The leaves are cleaned by washing using running water to remove the attached impurities, then the water bundle is dried using a tissue. 31

#### Metabolite Extraction

The size of the leaf sample was reduced using scissors and then weighed until it reached a weight of 138 g for extraction. Extraction of metabolites from mangrove leaves was performed by a single solvent maceration method using ethanol in a ratio of 1:2 (w/v) with several solvent changes due to solvent saturation. Samples were macerated with shaker agitation (100 rpm) for 24 hours at room temperature (25°C). After maceration, the organic solvent was taken and concentrated using a rotary evaporator at 35°C. The crude extract was then stored for subsequent analysis. 31

#### Metabolite Characterization

The characterization of the metabolites contained in the methanol extract was performed using phytochemical tests and liquid chromatography-high resolution mass spectrometry (LC-HRMS). Phytochemical tests were performed to detect the presence of compounds from the group of *alkaloids, flavonoids, saponins, steroids/triterpenoids, and tannins* . 31

Analysis of compounds using LC-HRMS was performed using two tools, namely liquid chromatography (LC) and mass spectrometry (MS). LC analysis was performed using the UltiMate™ 3000 RSLCnano System with a microflow meter (Thermo Scientific, USA). The column used was Hypersil GOLD aQ 50 × 1 mm × 1.9 µ particle size with a flow rate of 40 L/min for 30 minutes. The mobile phase used was 0.1% formic acid in water and 0.1% formic acid in acetonitrile. MS analysis was performed using Q Exactive Mass Spectrometers (Thermo Scientific, US). Compounds were screened with a resolution of 70.000 dab 17.000 for 30 minutes. The compound predictive analysis was performed using the mzCloud MS/MS Library.

### Antibacterial Diffusion Method

#### *Fusobacterium nucleatum* Culture and Preparation

*Fusobacterium nucleatum* (ATCC22586, UK) was cultured in the tryptic soy broth (TSB) media and incubated for 18 to 24 hours at 37°C under anaerobic conditions. Bacterial colonies were taken using a stick that was previously heated with a Bunsen burner then transferred in 3 mL of liquid brain heart infusion (BHI) media and incubated at 37°C for 18 hours. The bacterial suspension was equalized with the McFarland standard of 0.5 (1.5 × 10 ^9^ colony-forming unit [CFU]/mL). The suspension was then flattened on the surface of the nutrient agar medium.

#### *A. actinomycetemcomitans* Culture and Preparation

*A. actinomycetemcomitans* (ATCC43718, UK) cultures was incubated for 24 hours at 37°C under anaerobic conditions in the Brain Heart Infusion (BHI) media after taking out from the stock using a sterile stick. The cultures were matched to the McFarland standard of 0.5 or the equivalent of 1.5 × 10 ^8^ CFU/mL. The bacterial suspension was diluted once the turbidity of the bacterial suspension was not matched.

#### *P. gingivalis* Culture and Preparation

*P. gingivalis* (ATCC33277, UK) was cultured in TSB media and incubated for 18 to 24 hours at 37°C under anaerobic conditions. Bacterial colonies were taken using a stick that was previously heated over the Bunsen burner and then transferred in 3 mL of BHI liquid media and incubated at 37°C for 18 hours. The bacterial suspension was equalized with the McFarland standard of 0.5 (1.5 × 10 ^9^ CFU/mL). The suspension that was equalized was taken with a micropipette and then flattened on the surface of the nutrient agar media.

### Periodontopathogenic Bacteria Inhibition Zone Analysis

The inhibition zone was found in *F. nucleatum, P. gingivalis,* and *A. actinomycetemcomitans* culture plates after the administration of MLE with 100%, 75%, 50%, 25% as treatment groups and doxycycline as the positive group, respectively in the paper disk *.* The inhibition zone was calculated using a digital caliper (Mitutoyo, Japan) in a millimeter unit, then recapitulated for each group.

### Statistical Analysis

All research data were then recapitulated, analyzed descriptively, and inferentially. Data are presented as a mean and standard deviation that are presented in a bar chart. The data are analyzed using normality and homogeneity tests ( *p* > 0.05) together with the analysis of variance (ANOVA) difference test and the post-hoc Tukey honest significant different (HSD) with a different significance value of *p* < 0.05 using the statistical package for social science (SPSS) version 20.0 for Windows (IBM corporation, Illinois, Chicago, USA).

## Results

The ligand sample consisting of (-)-epicatechin, coumaric acid, and ascorbic Acid (vit C) was obtained with information on ID, formula, weight, and canonical smile. All the bioactive compounds contained in mangroves are classified as drug candidates based on Lipinski's predictions ( Table 1 ) and ADME-Tox ( Table 2 ). Protein samples consisting of HSP70, HSP30, HSP90, IL10, Foxp3, IL-6, peptidoglycan, flagellin, and dectin were obtained from the RCSB PDB database obtained with information on ID, visualization method, resolution, atom count, weight, chain, and sequence length ( Table 3 ).

**Table 1:** Results of ligand sample preparation and druglikeness

Compound	CID	Formula	SMILE	MW (Dalton)	LOGP	HBD	HBA	MR
(-)-Epicatechin	72276	C _15_ H _14_ O _6_	C1C(C(OC2 = CC(=CC(=C21)O)O)C3 = CC(=C(C = C3)O)O)O	290.000	1.546	5	6	72.622
Coumaric acid	1549106	C _9_ H _8_ O _3_	C1 = CC(=CC = C1C = CC(=O)O)O	164.000	1.490	2	3	44.776
Ascorbic Acid (vit C)	54670067	C _6_ H _8_ O _6_	C(C(C1C(=C(C(=O)O1)O)O)O)O	176.000	−1.407	4	6	35.256

Abbreviations: HBA, hydrogen bond acceptors; HBD, hydrogen bond donors; LOGP, high lipophilicity; MR, molar refractivity; MW, molecular mass.

**Table 2:** ADME-Tox analysis results

Compound	Water solubility (Log S)	Pharmacokinetics	Topological surface Area (Å)
(-)-Epicatechin	−2.24/Soluble	GI absorption: High Synthetic accessibility: 3.50	110.38
Coumaric acid	−1.83/Soluble	GI absorption: Low Synthetic accessibility: 4.30	136.88
Ascorbic Acid (vit C)	−0.10/Soluble	GI absorption: High Synthetic accessibility: 3.47	107.22

**Table 3:** Results of preparation of target protein samples from RCSB PDB

Name	PDB ID	Visualization Method	Resolution (Å)	Atom Count	Weight (kDa)	Chain	Sequence Length (mer)
HSP70	1S3X	X-Ray	1.84	3375	42.75	A	382
HSP30	1N3U	X-Ray	2.58	3666	55.14	A,B	233
HSP90	2XJX	X-Ray	1.66	1993	28.37	A	249
IL10	1INR	X-Ray	2.00	1114	18.67	A	160
Foxp3	4WK8	X-Ray	3.40	2243	33.05	C,D	82
IL-6	1IL6	NMR	−	166	21.01	A	185
Peptidoglycan	2OQO	X-Ray	2.10	1622	23.77	A	200
Flagellin	2ZBI	X-Ray	2.00	4198	60.96	A,B	292
Dectin	2CL8	X-Ray	2.80	2161	32.92	A,B	139

Based on the results of molecular docking simulations, (-)-epicatechin is one of the contents of mangrove extract that has the most negative binding energy compared to other compounds, it shows that (-)-epicatechin is predicted to affect the activity of target proteins such as inhibition and enhancement ( Table 4 ). Identification of molecular interactions and binding positions on the docked protein–ligand complex showed that the bonding of (-)-epicatechin compounds on all target proteins resulted in non-covalent bond interactions consisting of Vander Waals, pi, and hydrogen bonding ( Table 5 ).

**Table 4:** Molecular docking simulation results

Target	Ligand	Grid Positions		Binding Affinity (kcal/mol)
		Center	Dimensions	
HSP70	Coumaric acid	X: 17.346 Y: 32.160 Z: 13.643	X: 63.552 Y: 62.888 Z: 65.835	−6.4
	Ascorbic Acid			−6.9
	(-)-Epicatechin			−9.5
HSP30	Coumaric acid	X: 13.748 Y: 8.498 Z: −18.358	X: 79.021 Y: 74.334 Z: 92.834	−5.8
	Ascorbic Acid			−5.5
	(-)-Epicatechin			−7.8
HSP90	Coumaric acid	X: 17.346 Y: 28.675 Z: 16.057	X: 25.000 Y: 25.000 Z: 25.000	−6.5
	Ascorbic Acid			−6.9
	(-)-Epicatechin			−9.7
IL10	Coumaric acid	X: 13.0255 Y: 21.380 Z: 4.399	X: 45.583 Y: 38.549 Z: 66.373	−4.9
	Ascorbic Acid			−5.1
	(-)-Epicatechin			−6.5
Foxp3	Coumaric acid	X: −13.244 Y: −2.162 Z: −5.778	X: 61.474 Y: 57.400 Z: 57.492	−4.9
	Ascorbic Acid			−4.6
	(-)-Epicatechin			−6.2
IL-6	Coumaric acid	X: 13.025 Y: 21.380 Z: 4.399	X: 25.000 Y: 25.000 Z: 25.000	−4.5
	Ascorbic Acid			−4.2
	(-)-Epicatechin			−6.3
Peptidoglycan	Coumaric acid	X: 38.083 Y: 39.401 Z: 20.570	X: 66.196 Y: 48.507 Z: 55.925	−5.5
	Ascorbic Acid			−6.8
	(-)-Epicatechin			−7.5
Flagellin	Coumaric acid	X: −23.833 Y: 37.746 Z: 33.865	X: 116.859 Y: 44.857 Z: 97.698	−5.3
	Ascorbic Acid			−4.6
	(-)-Epicatechin			−8.4
Dectin	Coumaric acid	X: 44.987 Y: 23.784 Z: 44.486	X: 55.545 Y: 45.919 Z: 51.371	−5.4
	Ascorbic Acid			−5.4
	(-)-Epicatechin			−6.4

**Table 5:** Results of identification of molecular interactions

Molecular Complex	Molecular Interaction
(-)-Epicatechin-HSP70	Van der Waals: Gly12, Gly201, Gly12, Thr13 Hydrogen: Thr204, Gly203, Gly202, Thr14 Pi: Tyr15
(-)-Epicatechin-HSP30	Van der Waals: Gln38, Leu147, Phe214, Ala28, Phe207, Asn210, Thr135, Gly139, Glu29 Pi: Met34
(-)-Epicatechin-HSP90	Van der Waals: Gly338, Gly339, Arg272, Lys271 Hydrogen: Glu268, Asp366, Thr37
(-)-Epicatechin-IL10	Van der Waals: Glu50, Lys49, Leu46, Leu65, Leu101, Ile69 Hydrogen: Leu53
(-)-Epicatechin-Foxp3	Van der Waals: Arg414, Phe413, Leu351, Glu352, Arg347, Lys356, Glu354 Hydrogen: Arg347, Leu351 Pi: Trp348, Glu410
(-)-Epicatechin-IL-6	Van der Waals: Leu98, Tyr72, Met68 Hydrogen: Leu48
(-)-Epicatechin-Peptidoglycan	Van der Waals: Arg218, Val112, Gln113, Gly115 Hydrogen: Lys201, Ser116, Gly114, Thr82 Pi: Glu83, Arg100, Ala97, Arg85
(-)-Epicatechin-Flagellin	Van der Waals: Leu120, Asp171, Ser172, Leu173, Gln113, Asn393, Ala411, Asn174, Gly377 Hydrogen: Asn121, Thr117, Thr182, Gln176
(-)-Epicatechin-Dectin	Van der Waals: Thr117, Asp94, Asn86, Asp84 Pi: Lys396, Val378, Ala412

**Fig. 1:**
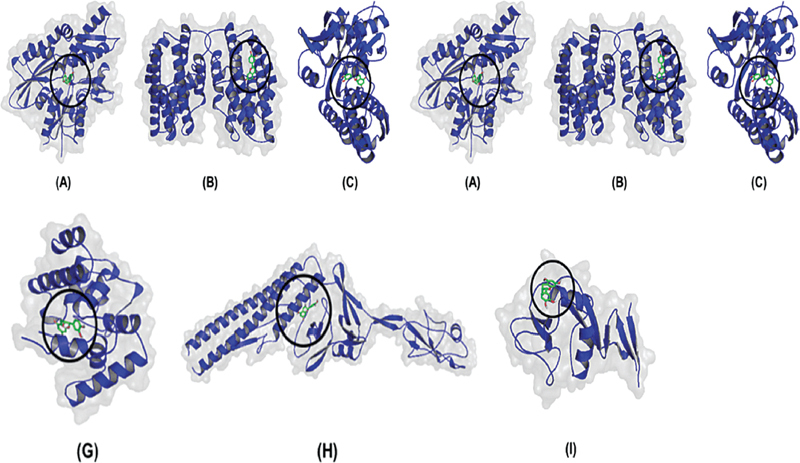
Three-dimensional (3D) visualization of docking results. ( **A** ) (-)-Epicatechin-HSP70 ( **B** ) (-)-Epicatechin-HSP30 ( **C** ) (-)-Epicatechin-HSP90 ( **D** ) (-)-Epicatechin-IL10 ( **E** ) (-)-Epicatechin-Foxp3 ( **F** ) (-)-Epicatechin-IL-6 ( **G** ) (-)-Epicatechin-peptidoglycan ( **H** ) (-)-Epicatechin-flagellin ( **I** ) (-)-Epicatechin-dectin.

All protein complexes that were docked were shown with cartoons structure, transparent surfaces, and colored selections based on their constituent structures, then docked for ligands displayed with sticks structure ( Fig. 1 ).

**Fig. 2:**
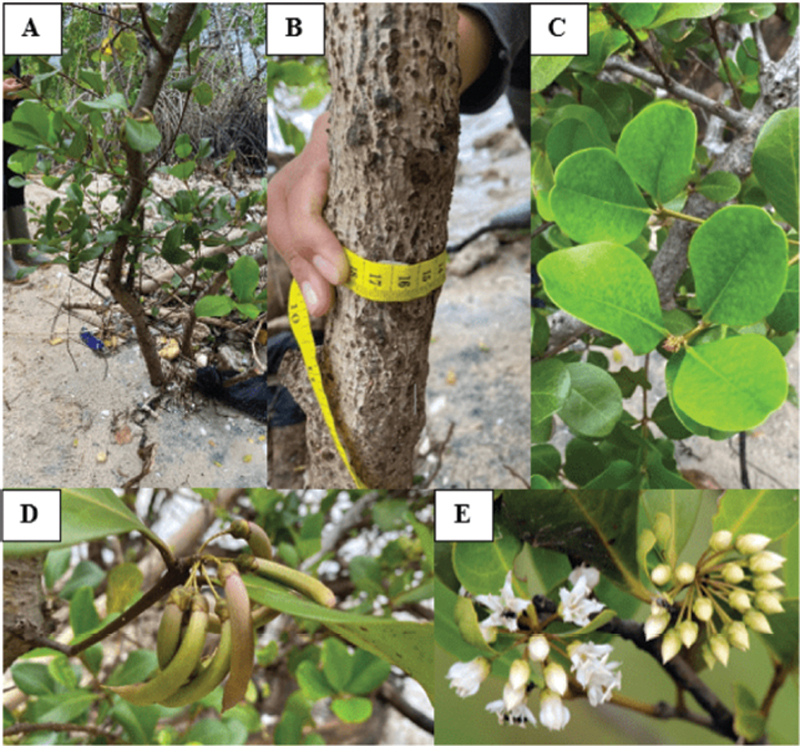
Morphology of mangrove samples ( **A** . Tree; **B** . Stem; **C** . Leaves; **D** . Fruit; **E** . Flowers).

Fig. 2 shows that the sample mangrove plants are in the form of small trees that grow straight and directly adjacent to the coastline. This mangrove trunk is gray-brown but most of the stems are buried by beach sand so that the root shape and total height are unknown. The leaves of this plant have fleshy skin, bright green in color, grow cross-legged, egg-shaped to elliptical and rounded ends, and some form a heart. The measurement results show that the mangrove leaves have an average length of 6.83 ± 0.77 cm and an average width of 4.61 ± 0.59 cm. The mangrove flower is white and has five petals and the overall shape is like a lantern. The characteristics of the sample plants are in accordance with the characteristics of the mangrove *A. corniculatum* , so that the sample used in this study is suspected to be mangrove *A. corniculatum* .

### Characteristics of Leaf Metabolites of *A. corniculatum*

Extraction using ethanol solvent gave a total crude extract yield of 2.69 g (1.94% w/w). This extract was then used for phytochemical tests and LC-HRMS. Phytochemical test results are shown in Table 6 .

**Tabel 6:** Phytochemical test results on *A. corniculatum* leaf extract

Analysis	Indicator	Result	Info
Alkaloid	Yellow to orange precipitate		+
Saponin	Stable foam		+
Flavonoid	Yellow/Orange/Red/Green		+
Triterpenoid and steroid	Terpenoids are red in the bottom layer, Steroids are green in the top layer		+
Tanin	Dark green/Blue green		+
Quinone	Reddish pink/Purple		−

**Fig. 3:**
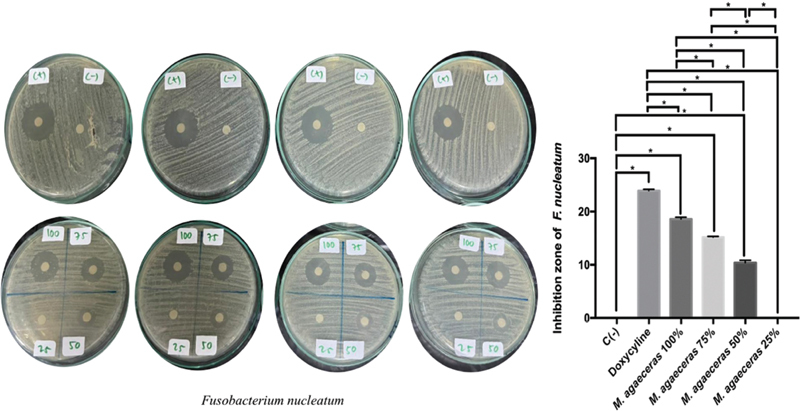
Periodontopathogen antibacterial inhibition zone of MLE against *F. nucleatum.* *Statistically significant *p* -value < 0.05.

In this study, it was found that 100% MLE has the ability to inhibit the growth of F. *nucleatum* ( Fig. 3 ). The most extensive zone of inhibition of *F. nucleatum* was found in doxycycline treatment followed by 100%, 75%, 50%, and 25%. There was a significant difference between the treatment groups on the inhibition zone of *F. nucleatum* . The inhibition ability of 100% MLE against *F. nucleatum* bacteria was higher than 75% ( *p* = 0.0001; *p* < 0.05).

**Fig. 4:**
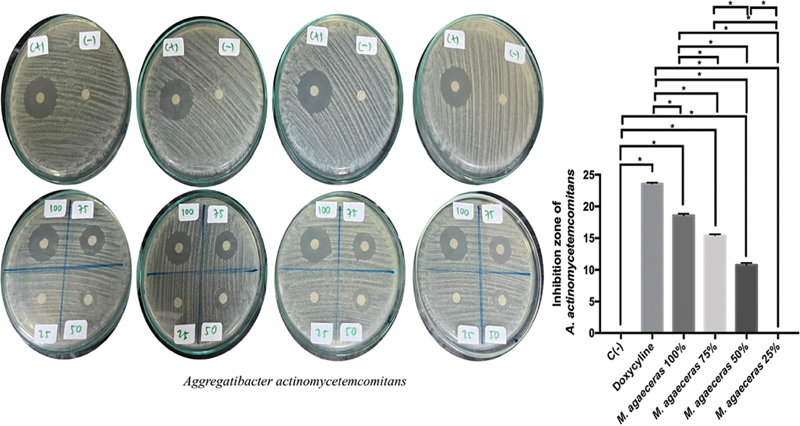
Periodontopathogen antibacterial inhibition zone of MLE against *A. actimomycetemcomitans.* *Statistically significant *p* -value < 0.05.

The inhibition growth of *A. actinomycetemcomitans* showed that 100% MLE had the highest ability to inhibit compared with others ( Fig. 4 ). The most extensive zone of inhibition of *A. actinomycetemcomitans* was found in doxycycline treatment followed by MLE 100%, 75%, 50%, 25%. There was a significant difference between the treatment groups on the inhibition zone of *A. actinomycetemcomitans* . The inhibition ability of 100% MLE against *A. actinomycetemcomitans* was higher than 75% MLE ( *p* = 0.0001; *p* < 0.05).

**Fig. 5:**
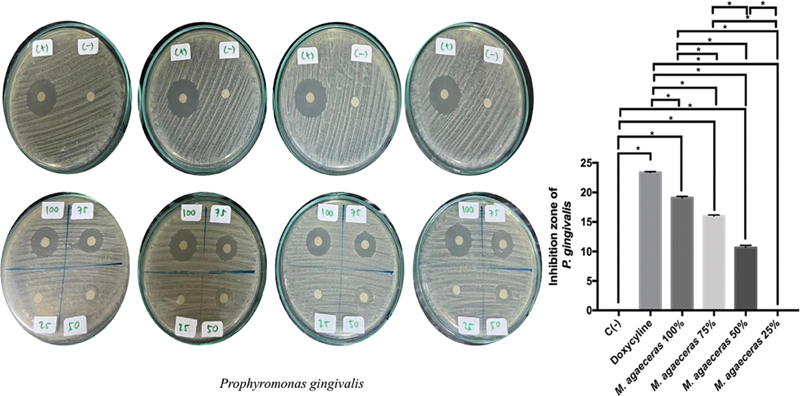
Periodontopathogen antibacterial inhibition zone of MLE against *P. gingivalis.* *Statistically significant *p* -value < 0.05.

100% MLE also shows the highest ability to inhibit the growth of *P. gingivalis* ( Fig. 5 ). The most extensive zone of *P. gingivalis* inhibition was found in doxycycline treatment followed by MLE 100%, 75%, 50%, 25%. There was a significant difference between the treatment groups in the inhibition zone of *P. gingivalis.* The inhibition ability of 100% MLE against *P. gingivalis* was higher than that of MLE 75% ( *p* = 0.0001; *p* < 0.05).

## Discussion

Humans have been using plants for the treatment of various infectious diseases since ancient times. Scientific research for proving the therapeutic efficacy of a large number of medicinal plants is undergoing. Today, medicinal plants are being used in many countries for the treatment of different infectious diseases. The current interest in medicinal plants as therapeutic agents has emerged in various parts of the world. Many scientific reports have shown potential foliar extracts of mangrove against microbial pathogens and suggested considering the mangrove plants as a valuable source for the bioactive chemicals of immense medicinal values. 32

One type of mangrove that is widely spread in various locations in Indonesia such as in Teluk Awur, Central Java is *A. corniculatum* . Mangrove has the potential as an antimicrobial against the causative agent of vibriosis. 21 Furthermore, mangrove ethanol extract obtained from mangroves grown on the coast of Mokupa Village, North Sulawesi has blood anticoagulant activity. 19 Furthermore, research on mangroves from various countries also proves that *A. corniculatum* has the potential as a source of anti-inflammatory, antiplasmodial, and antimicrobial compounds for human pathogens including *M. tuberculosis* . 18 20 22 23

Various compounds have been reported from mangrove *A. corniculatum* such as sakurasosaponin methyl ester, which has anticancer activity. Compounds 3-O-[α-L-rhamnopyranosyl-(1→2)-α-L-rhamnopyranosyl(1→2)-β-D-galactopyranosyl-(1→3)-β-D-glucopyranosyl(1→2)-β-D-(6′-O-methyl) glucuronopyranosyl]-13β,28epoxy-3β,16α-dihydroxy-olean, and sakurasosaponin *s* have been reported to be highly potential in triggering apoptosis of B16F10 melanoma cells. Compound (3β,16α,20α)-3,16,28-trihydroxyolean-12-en-29-oic acid3-{O-β-D-glucopyranosyl (1→2)-O-[β-D-glucopyranosyl (1→4)]-α-L-arabinopyranoside} and aegicoroside A have potential as anti-inflammatory agent. 20 33 2-hydroxy-5-ethoxy-3-nonyl1,4-benzoquinone and 5-O-butyl-embelin have also been reported as potential anticancer agents. 34 Although many active compounds have been reported from this mangrove, there have been no reports of active compounds from *A. corniculatum* from Indonesia.

This study reported that the MLE in 100% concentration has the most effective ability to inhibit *F. nucleatum, P. gingivalis, A. actinomycetemcomitans* growth in vitro. Alkaloid, saponin, flavonoid, triterpenoid, steroid, tannins, and quinone were detected in the MLE from phytochemical screening. Mechanism of action of *alkaloids* as an antibacterial is by way of interference with the components of the peptidoglycan of the bacterial cell and as a result, the lining of the cell walls are not fully formed which will lead to cell death and also by inhibiting the enzyme topoisomerase in bacterial cells and thus can strongly inhibit the nucleic acid synthesis and in turn bacterial growth. 35 36

The activity of *flavonoids* as an antibacterial agent is that they can damage the permeability of the cell walls of microbes. The functional protein binds to the DNA of cells thus can inhibit the growth of microbes. *Flavonoids* can inhibit cell components that function to release antimicrobial substances. The cell component lipopolysaccharide is found in the membrane of the cell. The mechanism of action of saponin as an antibacterial is that it can cause leakage of proteins and enzymes from within the cell. Because the surface-active ingredient saponin is similar to detergent, it reduces the surface tension of the bacterial cell wall and damage membrane permeability or leakage of cells, it resulting in discharge of intracellular compounds. 36 37

The mechanism of antibacterial action of the *triterpenoids* is that they act by disrupting the cytoplasmic membrane by inhibiting peptidation of the growing peptidoglycan chain and also inhibiting enzymes for cell wall synthesis. 38 Steroid compound that can be found in the herbal extract may possessed antibacterial activity. Steroid may enhance the intermolecular interaction via divalent cation such as MG2 and CA2 that increase the gram negative bacteria outer membrane cell permeability trigger the inhibition of gram negative bacterial growth. 39

The antibacterial effectiveness of *tannins* is explained by their ability to pass through the bacterial cell wall up to the internal membrane, interfere with the metabolism of the cell, and as a result, cause their destruction. In addition, tannins inhibit the bacterial attachment to the surface resulting in bacterial cell death. Moreover, the sugar and amino acid uptake are inhibited by tannic acid which limits the bacteria growth. 40 *Quinon* as an antibacterial that acts by forming irreversible complexes with amino acids in proteins, which lead to their inactivation. Thus, they target cell wall constituents including surface-exposed adhesins, cell wall polypeptides, and membrane-bound enzymes. 41

The activity level of (-)-epicathecin as a compound of mangrove extract showed that it can act as an antioxidant compared with ascorbic acid and coumaric acid through increased expression of HSP30, HSP70, and HSP90. (-)-Epicatechin is predicted to act as an anti-inflammatory agent compared with ascorbic acid and coumaric acid with the mechanism of increasing FOXP3 expression and IL-10 secretion. In addition, the anti-inflammatory response produced by (-)-epicatechin occurs because it initiates the inhibitory pathway of IL-6 expression. Then, the antibacterial activity was also produced by (-)-epicatechin compounds compared with ascorbic acid and coumaric acid, this was indicated by the possibility of inhibition by (-)-epicatechin on peptidoglycan, flagellin, and dectin on bacterial membranes.

Free radicals and inflammation are linked, as free radicals are the crucial signaling components that lead to membrane dysfunction as well as tissue damage at the inflammation site. During inflammation, oxidative stress causes the opening of inter-endothelial junctions to allow the migration of inflammatory cells. 42 The antioxidants that have the potential to suppress the NADPH oxidase-dependent O _2_^−^ generation might serve as an effective anti-inflammatory agent. During inflammatory conditions, NADPH oxidase residing in polymorphonuclear and mononuclear cells are activated and generate ROS. ROS are among the most potent stimuli responsible for increasing vascular permeability, enhancing the production of proinflammatory cytokines (TNF-a, IL-8, IL-1), chemotactic factors, and provoking lipid peroxidation (plasma membrane) and oxidation of DNA. Thus, ROS deregulate the cellular function and induce tissue damage, which in turn augments the state of inflammation. 43

Non-enzymatic antioxidants include polyphenolic compounds, such as phenols, tannin, and flavonoids. It has been proposed that flavonoids and polyphenolic compounds act as reducing agents either as enzyme cofactors or electron donors. Generally, phenolic flavonoids and tannins are mostly associated with the antioxidant activity of plants. *A. corniculatum* bark is a potent antioxidant (IC50 20.49 ± 2.14 μg/mL in DPPH assay) with anti-inflammatory (IC50 23.58 ± 1.75 μg/mL in LOX inhibition assay). 42 Limitations of this study are the limited bioactive content of MLE used in molecular docking as well as bacterial strains used in *in vitro* studies.

## Conclusion

The active compound from Indonesian MLE ( *Aegiceras corniculatum* ) was investigated for developing a herbal-based mouthwash through *in silico* and in vitro studies. However, further study is still urgently needed to investigate the mangrove leaf extract *in vivo* with various methods. A complex study should be conducted to elucidate the complex mechanism of how mangrove extract can promote the regeneration of periodontal tissue.
